# Establishment of a prognostic model for hypoxia-associated genes in OPSCC and revelation of intercellular crosstalk

**DOI:** 10.3389/fimmu.2024.1371365

**Published:** 2024-06-03

**Authors:** Yichen Zhao, Jintao Yu, Chang Zheng, Baosen Zhou

**Affiliations:** Department of Clinical Epidemiology and Center of Evidence-Based Medicine, The First Hospital of China Medical University, Shenyang, China

**Keywords:** hypoxia, HIF-1α, oropharyngeal squamous cell carcinomas, human papillomavirus, single-cell

## Abstract

Hypoxia exerts a profound influence on the tumor microenvironment and immune response, shaping treatment outcomes and prognosis. Utilizing consistency clustering, we discerned two hypoxia subtypes in OPSCC bulk sequencing data from GEO. Key modules within OPSCC were identified through weighted gene correlation network analysis (WGCNA). Core modules underwent CIBERSORT immune infiltration analysis and GSEA functional enrichment. Univariate Cox and LASSO analyses were employed to construct prognostic models for seven hypoxia-related genes. Further investigation into clinical characteristics, the immune microenvironment, and TIDE algorithm prediction for immunotherapy response was conducted in high- and low-risk groups. scRNA-seq data were visually represented through TSNE clustering, employing the scissors algorithm to map hypoxia phenotypes. Interactions among cellular subpopulations were explored using the Cellchat package, with additional assessments of metabolic and transcriptional activities. Integration with clinical data unveiled a prevalence of HPV-positive patients in the low hypoxia and low-risk groups. Immunohistochemical validation demonstrated low TDO2 expression in HPV-positive (P16-positive) patients. Our prediction suggested that HPV16 E7 promotes HIF-1α inhibition, leading to reduced glycolytic activity, ultimately contributing to better prognosis and treatment sensitivity. The scissors algorithm effectively segregated epithelial cells and fibroblasts into distinct clusters based on hypoxia characteristics. Cellular communication analysis illuminated significant crosstalk among hypoxia-associated epithelial, fibroblast, and endothelial cells, potentially fostering tumor proliferation and metastasis.

## 1 Introduction

Head and neck squamous cell carcinomas (HNSCCs) emerge from the mucous membrane of the oral cavity, pharynx, and larynx, and are the most prevalent malignancies of the head and neck (1). With a 5-year survival rate of only 40–50% (2), HNSCC frequently receives a diagnosis in a late stage when it is challenging to cure. Although tobacco (3) and alcohol used (4), betel quid chewing (5), and poor oral hygiene (6) are still strongly associated with HNSCC, high-risk oncogenic human papillomavirus (HPV) types, particularly HPV type 16, plays a major role in the pathogenesis and progression of HNSCC (7). HPV viruses are known to be causally linked to a subset of oropharyngeal squamous cell carcinomas (OPSCC) arising from the palatine and lingual tonsils (8).

HPV16 expresses E6 and E7 proteins. The E6 oncoprotein disrupts the p53 pathway, leading to cell cycle dysregulation, and the E7 oncoprotein induces retinoblastoma protein inhibition, transforming infected cells into cancer cells (9). Patients with HPV-positive OPSCC typically respond well to treatment and have better long-term survival than patients with HPV-negative OPSCC and HNSCC in general, regardless of whether they receive radiotherapy alone or radio-chemotherapy (10).

Hypoxia is a hallmark of the tumor microenvironment (TME) in major human tumors and promotes tumor malignancy, causing resistance to radiation therapy, immune evasion and immune resistance (11). HIF-1α (hypoxia-inducible factor 1-α) is a transcription factor that is essential for cells to cope with low oxygen levels, regulating genes involved in metabolism, angiogenesis and survival under hypoxic conditions (12). Earlier studies have shown that cells maintaining the HPV genome display elevated levels of HIF-1α and that E7 enhances HIF-1α transcriptional activity (13, 14). However, HPV-associated OSCC exhibits lower levels of tumor hypoxia, which may be related to the unique intrinsic ability of HPV-positive tumor cells to adapt to hypoxia and their better prognosis (15). In recent years, the rise of single-cell RNA sequencing (scRNA-seq) technology (16) has become a powerful tool for enabling an understanding of the heterogeneity of the tumor microenvironment and mechanisms of cancer progression. Still, studies using methods combining bulk sequencing with single-cell sequencing in relation to hypoxia-associated subtype characterization, tumor immune landscapes, and intercellular communication remain rare.

This study centers on the subset of head and neck cancer primarily linked to HPV, namely OPSCC. Initial scrutiny involved the analysis of distinct phenotypes arising from hypoxia-associated genes across various bulk sequencing datasets. Hypoxia models and corresponding risk scores were formulated with prognostic evaluations. Subsequent investigations delved into diverse hypoxia phenotypes and associated risks, with a specific focus on their interplay with HPV status, immunological attributes, and implications for immunotherapy outcomes. Then we employed the scissor algorithm in conjunction with single-cell sequencing datasets to obtain subpopulations of cells associated with hypoxia, and further did cellular communication and metabolic analyses. This integrated approach is intended to aid in personalizing treatment strategies and to facilitate the elucidation of new therapeutic targets, providing insights for advancing tailored therapeutic interventions.

## 2 Materials and methods

### 2.1 Data collection and processing

**Figure 1:**
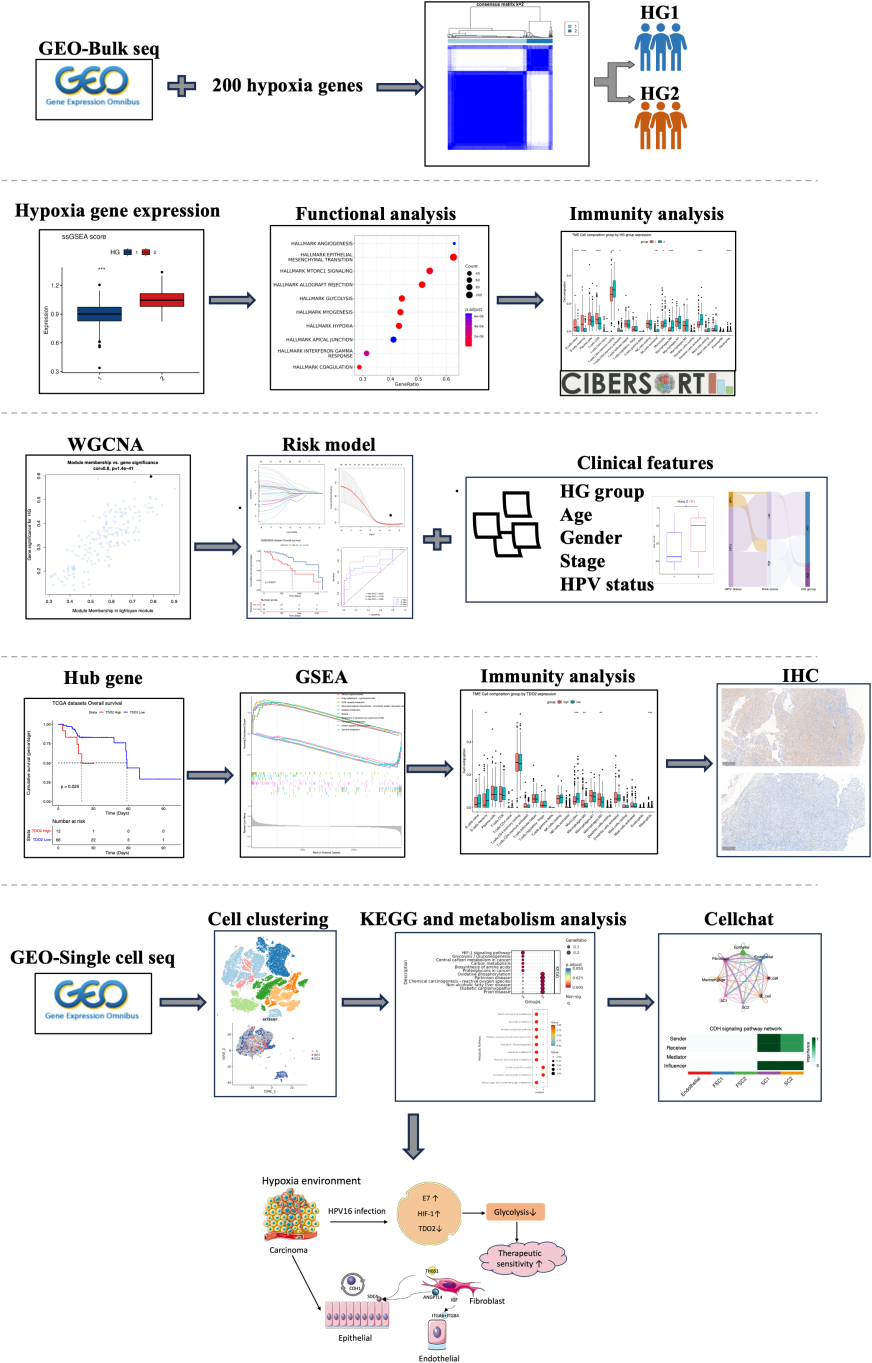
Flowchart of this study.

The sequencing data and microarray data for this study were derived from the Gene Expression Omnibus (GEO) database (http://www.ncbi.nlm.nih.gov/geo/), in which GSE171898 (262 HPV+,49HPV-) and GSE65858 (**Supplementary Table 1**) were selected as the primary datasets, and specifically extracted from the HPV containing information from OPSCC samples. In addition, as a validation dataset, we downloaded transcripts per kilobase million (TPM) data of HNSC from The Cancer Genome Atlas (TCGA) database (https://tcga-data.nci.nih.gov/tcga/) and screened 70 OPSCC samples from it (**Supplementary Table 2**). For single-cell sequencing data, we obtained them from the GSE182227 dataset of the GEO database. This dataset covers a total of 70,970 cells from 11 HPV-positive and 5 HPV-negative oropharyngeal squamous cancer patients. We used the ‘Seurat’ package (version: 4.4.0) to read the raw sequencing data. During the data processing stage, cells with a total gene count of less than 200, cells with a mitochondrial gene proportion of more than 20%, and cells with a hemoglobin gene proportion of more than 1% were filtered out. ‘SCP’ R package (version:0.5.6, https://github.com/zhanghao-njmu/SCP.), a single-cell data-processing package based on the Seurat algorithm, was used to perform visualization and enrichment analysis of the results (**Figure 1**).

### 2.2 Consistency clustering of hypoxia-related genes

The 200 hypoxia-associated genes were obtained from the hallmarks gene set of the GSEA database. Consistency clustering was used with the ‘ConsensusClusterPlus’ package (version:1.60.0). Single-sample gene set enrichment analysis (ssGSEA) was used to quantify hypoxia score in each sample using the ‘GSVA’ R package (version:1.44.0).

### 2.3 WGCNA analysis

The weighted gene co-expression network analysis (WGCNA) R package (version:1.71) was analyzed using weighted gene co-expression network to obtain critical genes after hypoxia clustering. Based on the GSE171898 expression matrix, the proximity matrix was transformed into a topological overlap matrix by choosing an appropriate power index. Then correlation analysis between gene co-expression modules and hypoxia group (HG) phenotypes was performed, and the modules positively correlated with HG and with the highest correlation were selected for further analysis.

### 2.4 Immune infiltration analysis

CIBERSORT (https://cibersortx.stanford.edu/) was used to assess the proportions of 22 immune-infiltrating cell types in each sample in different hypoxia groups and different HPV groups. After removing samples with p-values < 0.05, the empirical p-value of the back-convolution in each case was determined. In addition, the Tumor Immune Dysfunction and Exclusion (TIDE) algorithm (http://tide.dfci.harvard.edu/) was used to predict the response to immune checkpoint inhibitors in patients with OPSCC.

### 2.5 Functional enrichment analysis

Gene set enrichment analysis (GSEA) was performed using the ‘ClusterProfiler’ (version: 4.10.0) package to explore the differences in function and associated pathways between the HG1 and HG2 groups. The groups were categorized into low and high expression groups based on differential gene expression. The C2 (KEGG), C5 (GO), C6 (cellular pathway), C7 (immune pathway) gene sets downloaded from the Molecular Marker Database (MsigDB) (https://www.gsea-msigdb.org/gsea/msigdb/) were used as reference gene sets. The same was visualized with the ‘ClusterProfiler’ package.

### 2.6 Construction of hypoxia score

The GSE65858 data was utilized as a training set to construct the model. Module genes with the highest correlation with hypoxia phenotype obtained from WGCNA analysis were selected. Univariate Cox proportional risk regression analysis was performed on the candidate genes in the training set to screen for characteristic genes associated with prognosis. Significant variables were included in the Least absolute shrinkage and selection operator (LASSO) regression analysis, which was performed with the R software ‘glmnet’ package to reduce the number of genes in the final risk model according to the risk score = *gene exp1 × β1 + gene exp2 × β2 + … + gene expression n × βn*. Patient survival curves and risk maps were visualized using the ‘survminer’ and ‘ggrisk’ packages. ROC curves were plotted using the ‘timeROC’ package (version:0.4) to assess the performance of OPSCC patients in predicting overall status (OS) risk scores at 1, 3 and 5 years. In addition, an external dataset, TCGA-OPSCC, validated the predictive model. Gene Set Variation Analysis (GSVA) was used to determine the risk score in different hypoxia groups via ‘GSVA’ R package (version:1.44.0).

### 2.7 Scissors algorithm for identifying phenotype-associated cells

Scissor algorithm (17) is a novel method for analyzing single-cell data. Bulk phenotypes are utilized to identify subpopulations of cells highly correlated with phenotypes from single-cell sequencing data. The hypoxia phenotype was chosen as the phenotype for the logit model. The parameter alpha was set to 0.2 to identify the most relevant hypoxic subtype.

### 2.8 Cell Communication analysis

Explore interactions between cell clusters using the ‘CellChat’ (version:1.6.1) software package (18). The tool is based on 2021 CellChatDB experimentally validated ligand-pair predictions of cellular interactions in the database. Interactions and interaction strengths between different cell subpopulations were calculated and the overall information flow of each signaling pathway was compared.

### 2.9 Single-cell hypoxia scoring and metabolic detecting

Use ‘AUCell’ (version: 1.24.0) to identify cells with a hypoxia-active gene set in single-cell RNA data. AUCell uses Area Under the Curve (AUC) to calculate whether a critical subset of the input gene set is enriched in expressed genes in each cell. First, we use the ‘AUCell _ buildRankings’ function to calculate the gene expression rankings in each cell using an expression matrix for each cell using default parameters. ‘AUCell_exploreThresholds’ was used to determine the threshold for gene set activity. We used ‘scMetabolism’ (version: 0.2.1) (19) to analyze differences in metabolic pathway activity in the single-cell dataset. ‘scMetabolism’ is an R package based quantification of metabolic activity at the single-cell level. It uses the VISION algorithm to score each cell, ultimately obtaining an activity score for each cell in each metabolic pathway.

### 2.10 Patients’ characteristics and specimens’ collection

Tissues were collected for immunohistochemical validation from 46 patients operated for oropharyngeal squamous carcinoma at the Department of Otorhinolaryngology Head and Neck Surgery, the First Affiliated Hospital of China Medical University between 2013 and 2017. The patient cohort ranged in age from 25 to 75 years, consisting of 7 females and 39 males. Clinical data, including patient age, gender, and tumor pathological grading, were meticulously recorded for further investigation. Detailed information is presented in **Supplementary Table 1**.

### 2.11 Immunohistochemical experiment validation

Immunohistochemistry was used to evaluate P16 and TDO2 in oropharyngeal cancers. Positive staining of P16 protein was defined as HPV positive. All tissues were paraffin-embedded, fixed and serially sectioned for use. After elimination of endogenous enzymes with 3% peroxidase solution, tissues were incubated in 5% bovine serum albumin for 20 minutes at room temperature and then incubated overnight with primary antibodies (anti-CDKN2A,P16: Abcam; anti-TDO2: Proteintech). The next day, the tissues were incubated with the secondary antibody for 30 min and visualized with 3,3′-diaminobenzidine. Finally, the slides were counterstained with hematoxylin, dehydrated, and cover-slipped. The immunohistochemical scoring was conducted by experienced pathologists using a semi-quantitative method, simultaneously assessing staining intensity and the number of positive cells. Different percentages of positive cells corresponded to different scores: 0%–25% for 1 point, 26%–50% for 2 points, 51%–75% for 3 points, and 76%–100% for 4 points. Different staining intensities corresponded to different scores: no staining for 0 points, pale yellow for 1 point, light brown for 2 points, and dark brown for 3 points. The sum of scores for positive cell percentage and staining intensity ranged from 1 to 7. Scores less than 3 were considered negative expression, while scores greater than 3 were considered positive expression. This study was approved by the Ethics Committee of the First Affiliated Hospital of China Medical University (No.[2022]199) and conformed to the Declaration of Helsinki.

### 2.12 Statistical analysis

All statistical data were based on R software (version: 4.3.1). In this study, we used t-test and one-way ANOVA to compare continuous variables. The χ^2^ test was employed to assess the statistical significance of the relationship between TDO2 expression and clinicopathological variables. Correlation and Wilcoxon rank sum test were used to compare the infiltration of immune cells in the TDO2 high and low expression groups. Disease-free survival was defined as the duration from diagnosis to the occurrence of the initial local recurrence or metastasis. Overall survival was defined as the period from the date of diagnosis to the date of death or to the latest follow-up. The survival rate data were analyzed using the Kaplan-Meier method, with survival curves compared utilizing the log-rank test at a significance level of 0.05. Univariate regression was conducted to identify hypoxia-related genes associated with prognosis, followed by further selection of core genes using Lasso Cox regression. Multivariate regression analysis was performed to ascertain the prognostic factors of clinical characteristics, including age, gender, HPV status, clinical stage, smoking, alcohol consumption, and risk score. Hazard ratios and their corresponding 95% confidence intervals were computed to estimate risk. Statistical significance was defined as a p-value less than 0.05.

## 3 Results

### 3.1 Unsupervised clustering of hypoxia-associated genes reveals two distinct classes with varied immune and glycolytic activity

**Figure 2:**
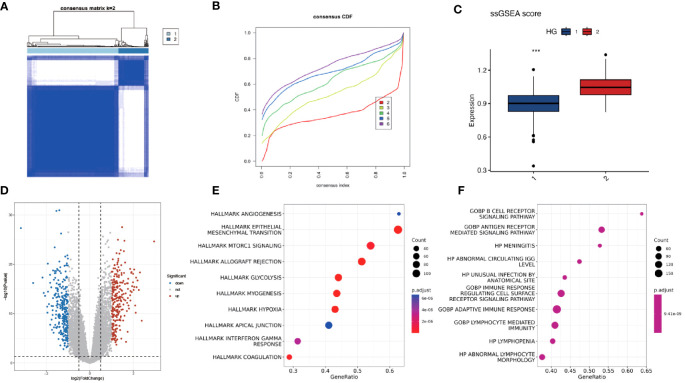
Consistency clustering of hypoxia-associated genes revealed two distinct classes with varying levels of immune and biological function. **(A, B)** Consensus matrix of 311 OPSCC samples based on 200 hypoxia-related genes. Two subtypes were determined for all samples (k = 2). **(C)** ssGSEA analysis of expression abundance of 200 hypoxia genes in different HG groups (1-HG1, 2-HG2). **(D)** Volcano plot of DEGs between HG1 and HG2 groups. P < 0.05 and |log2FoldChange|>1 were identified as significant DEGs. The red dots represent upregulated genes and the blue dots represent downregulated genes. **(E, F)** Bubble plots of the Hallmark and GO pathways of DEGs. *** means p<0.001.

**Figure 3:**
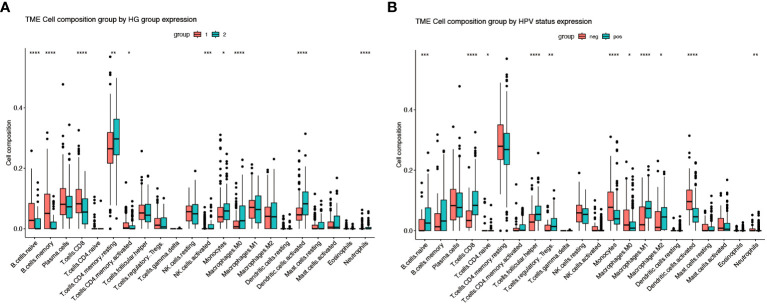
Immune infiltration of different gene subgroups. **(A)** The fractions of 22 immune cells between the HG1 and HG2 by the CIBERSORT method (group 1-HG1, group 2-HG2). **(B)** The fractions of 22 immune cells between the HPV-positive and HPV-negative groups by the CIBERSORT method (group neg-Negative, group pos-Positive). * Means *p*<0.05; ** means *p*<0.01; *** means *p*<0.001; **** means *p*<0.0001.

Using unsupervised clustering of 200 hypoxia-associated genes in 311 samples from GSE171898, we determined that the samples were optimally classified into two classes: HG1 and HG2 (**Figures 2A–C**). To further identify other phenotypic differences caused by hypoxic patterns, 523 differential genes were found, 256 up-regulated and 267 down-regulated (*logFC*>1, *P*<0.05) (**Figure 2D**). The GSEA functional enrichment analysis revealed that these genes were predominantly enriched in hallmark hypoxia, hallmark glycolysis, GOBP adaptive immune response, GOBP immune response regulating cell surface receptor signaling pathway. Subsequent ssGSEA analysis of the HG1 and HG2 classes for hypoxia-related gene expression indicated higher levels in the HG2 class (**Figures 2E, F**). CIBERSORT analysis of immune cell infiltration across the hypoxia classes and HPV status revealed divergent patterns, with most infiltrates being more abundant in the HG1 class compared to the HG2 class (**Figures 3A, B**). Recognizing WGCNA as a robust method for detecting key genes associated with specific modules, we utilized this approach to identify genes significantly correlated with HG. Initially, we selected 4 as the soft-thresholding power (**Supplementary Figures 1A, B**). Among them, the lightcyan and midnight blue modules showed a significant positive correlation with HG. The light cyan module, which exhibited the highest correlation (*cor* = 0.8, *P* < 0.05), was chosen for subsequent analysis (**Supplementary Figures 1C, D**).

### 3.2 Construction of hypoxia model genes

**Figure 4:**
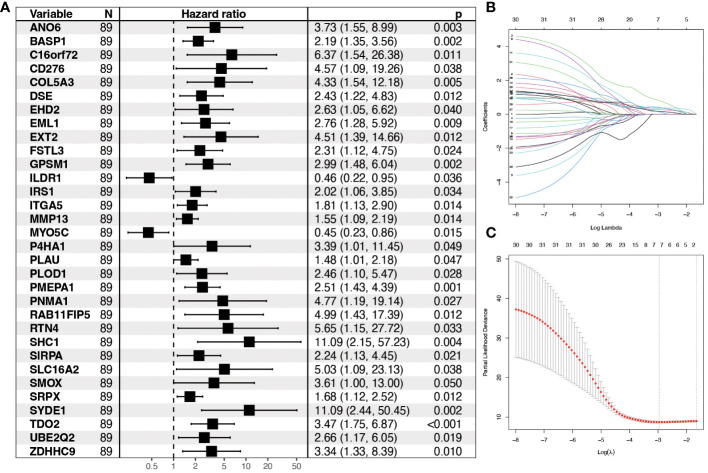
Construction of prognostic model for hypoxia-related genes. **(A)** Forest plot showing Hazard ratio and 95% CI derived from univariate Cox regression analysis of significant genes in the lightcyan module of WGCNA. HR for each variable is depicted as a box, and 95% CIs are shown as horizontal lines. The vertical line crossing the value of 1 represents a non-statistically significant effect, and odds greater than one indicate worse effects. **(B)** LASSO coefficient distribution of each independent gene. **(C)** The partial likelihood deviance in LASSO Cox regression analysis.

**Figure 5:**
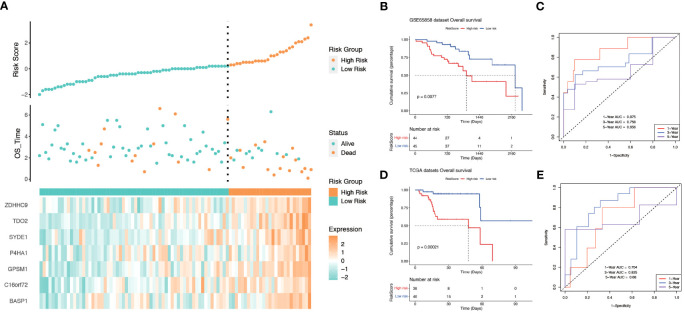
The relationship between risk score and survival. **(A)** Relationship between survival status and risk score in GSE65858 cohort. **(B)** Kaplan−Meier curves for OS(Overall Survival) for different risk score groups in the GSE65858 cohort. **(C)** ROC curves of key risk genes for predicting 1-, 3-, and 5-year OS in the GSE65858 cohort. **(D)** Kaplan−Meier curves for OS(Overall Survival) for different risk score groups in TCGA-OPSCC. **(E)** ROC curves of key risk genes for predicting 1-, 3-, and 5-year OS in the TCGA cohort.

All genes within the lightcyan module underwent prognostic correlation analysis and gene screening within the GSE65858 dataset, which contains prognostic information. Univariate Cox regression analysis identified 32 hypoxia-related genes significantly associated with overall survival (**Figure 4A**). Core genes were further refined using LASSO-Cox regression, resulting in a prognostic model comprising seven genes that became more robust with an increased penalty (**Figures 4B, C**). These genes are BASP1, C16orf72, GPSM1, P4HA1, SYDE1, TDO2, and ZDHHC9. A prognostic model utilizing risk scoring was established based on the gene expression levels and regression coefficients of seven genes and each patient was assigned a risk score. Using the median risk score as the threshold, patients in each dataset were classified into low-risk and high-risk groups. Overall survival significantly differed between the two groups (P = 0.0077, log-rank test). **Figures 5A** and **B** shows the higher scores suggest a worse prognosis. We further performed external validation using TCGA-OPSCC, which showed that the model also had some predictive power (**Figure 5D**). ROC curves were generated using the timeROC package (**Figures 5C, E**). To discern whether the risk score could have independent prognostic assessment ability, we collated clinical information of patients and filtered these clinical factors by multifactorial Cox stepwise regression, and we found that hypoxic risk score and stage were independent risk factors for patient prognosis. Finally, we used the clinical information and risk model to build a prognostic correlation prediction nomogram (**Supplementary Figures 2A–C**).

### 3.3 Association between immune infiltration and hypoxia score

**Figure 6:**
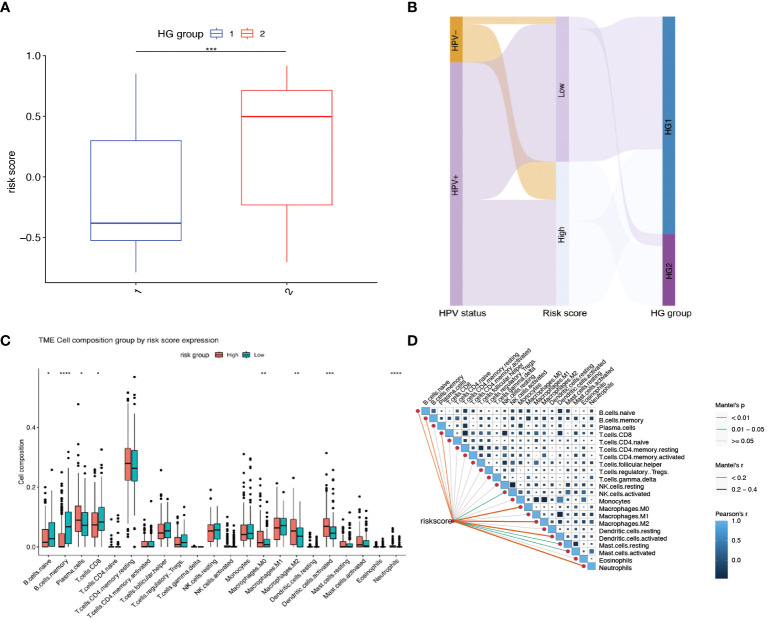
Relationship between risk scores and different hypoxia groups, HPV status, and immune cell infiltration. **(A)** Gene Set Variation Analysis (GSVA) to calculate risk scores for different hypoxia groups. (HG1:N=234, HG2:N=77) **(B)** Sankey diagram showing the distribution of patients with HPV status, risk score and HG group. **(C)** The fractions of 22 immune cells between the high and low risk groups by the CIBERSORT method. **(D)** Correlations between risk score and the abundance of each immune cell in 311 OPSCC samples. * means p<0.05; ** means p<0.01; *** means p<0.001; **** means p<0.0001.

**Figure 7:**
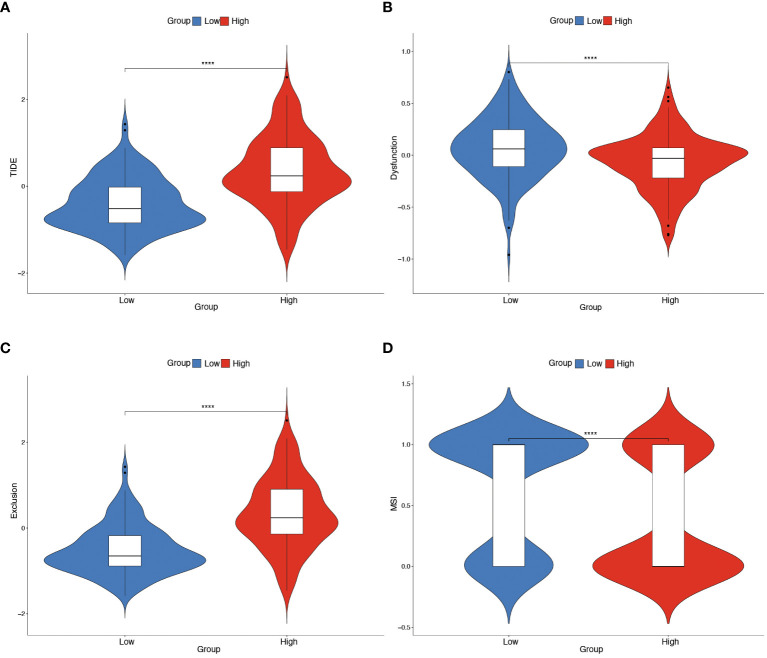
Predicting immunotherapy efficacy based on risk scores. **(A–D)** Differences in TIDE, T-cell dysfunction score, T-cell exclusion score and MSI in the two risk score subgroups. The difference between positive and negative groups was compared through the Wilcoxon rank-sum test. **** means *p*<0.0001.

Further investigation was conducted to explore the relationship between hypoxia phenotype, risk score, and HPV status. The HG2 risk score was higher (**Figure 6A**) and most HPV16-positive patients were in the HG1 and low-risk groups (**Figure 6B**). Increasing evidence reveals that the hypoxic microenvironment may protect tumors from natural anti-tumor immune responses by inhibiting anti-tumor immune effector cells and promoting immune escape mechanisms. The CIBERSORT method combined with the LM22 feature matrix was used to assess the differences in immune infiltration of 22 immune cells in patients in the high and low risk groups of oropharyngeal cancer (**Figure 6C**). Plasma cells, M0 macrophages, M2 macrophages, activated dendritic cells, and neutrophils showed relatively high infiltration in the high-risk group. In contrast, naïve B cells, memory B cells, and CD8+ T cells were infiltrated to a relatively low extent in the high-risk group. Correlation analysis showed a high correlation between immune cell subsets (e.g., memory B cells, CD8+ T cells, activated NK cells, etc.) and risk scores (**Figure 6D**). In the GSE17189 cohort, the TIDE score was significantly higher in the high risk score group than in the low score group (**Figure 7A**). In addition, there were significant differences in T-cell dysfunction scores (**Figure 7B**) and T-cell exclusion scores (**Figure 7C**) in addition to MSI scores (**Figure 7D**) between the two groups. These results suggest that patients with high hypoxia scores have poor immunotherapy benefit, which is consistent with previous findings.

### 3.4 Prognostic impact of TDO2 expression and its association with HPV status in OPSCC

**Figure 8:**
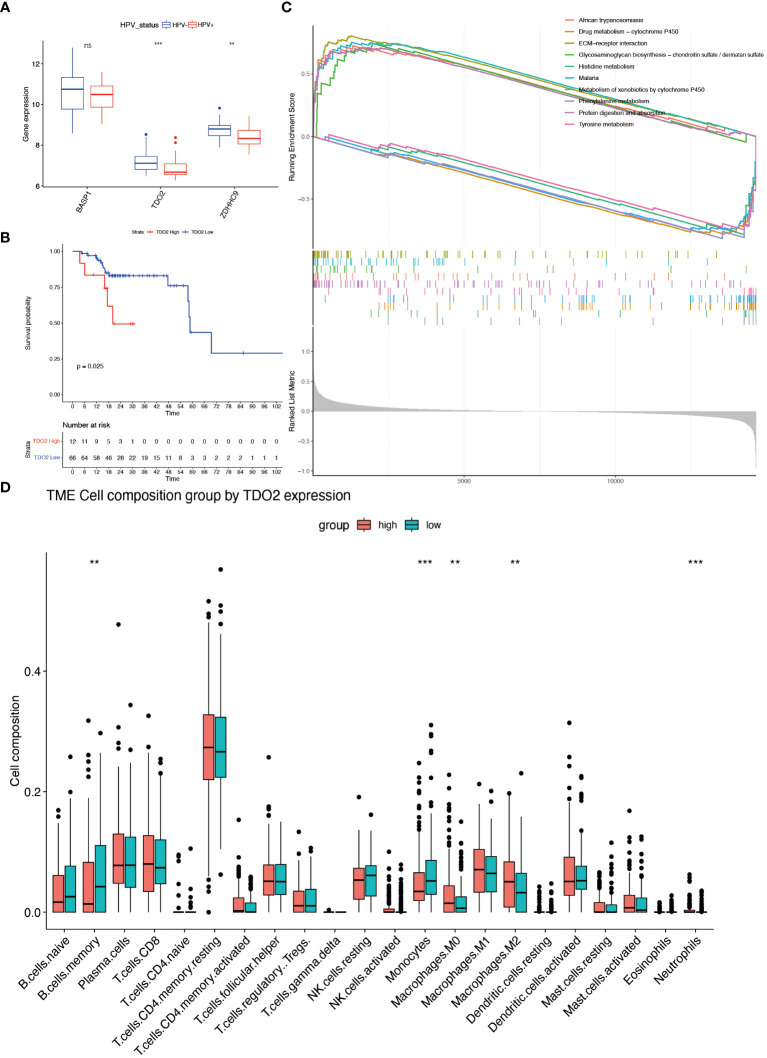
Characterizations of hubgene. **(A)** Differential expression of BASP1, TDO2 and ZDHHC3 in different HPV status. **(B)** Kaplan-Meier analysis of patients in the high and low TDO2 expression group in TCGA-OPSCC cohort (Grouped according to the best cut-off value). **(C)** GSEA enrichment analysis of the GSE65858 dataset based on high and low TDO2 expression. **(D)** The fractions of 22 immune cells between the high- and low-expression groups of TDO2 by the CIBERSORT method. ** means *p*<0.01; *** means *p*<0.001; **** means *p*<0.0001. ns means none significance.

**Figure 9:**
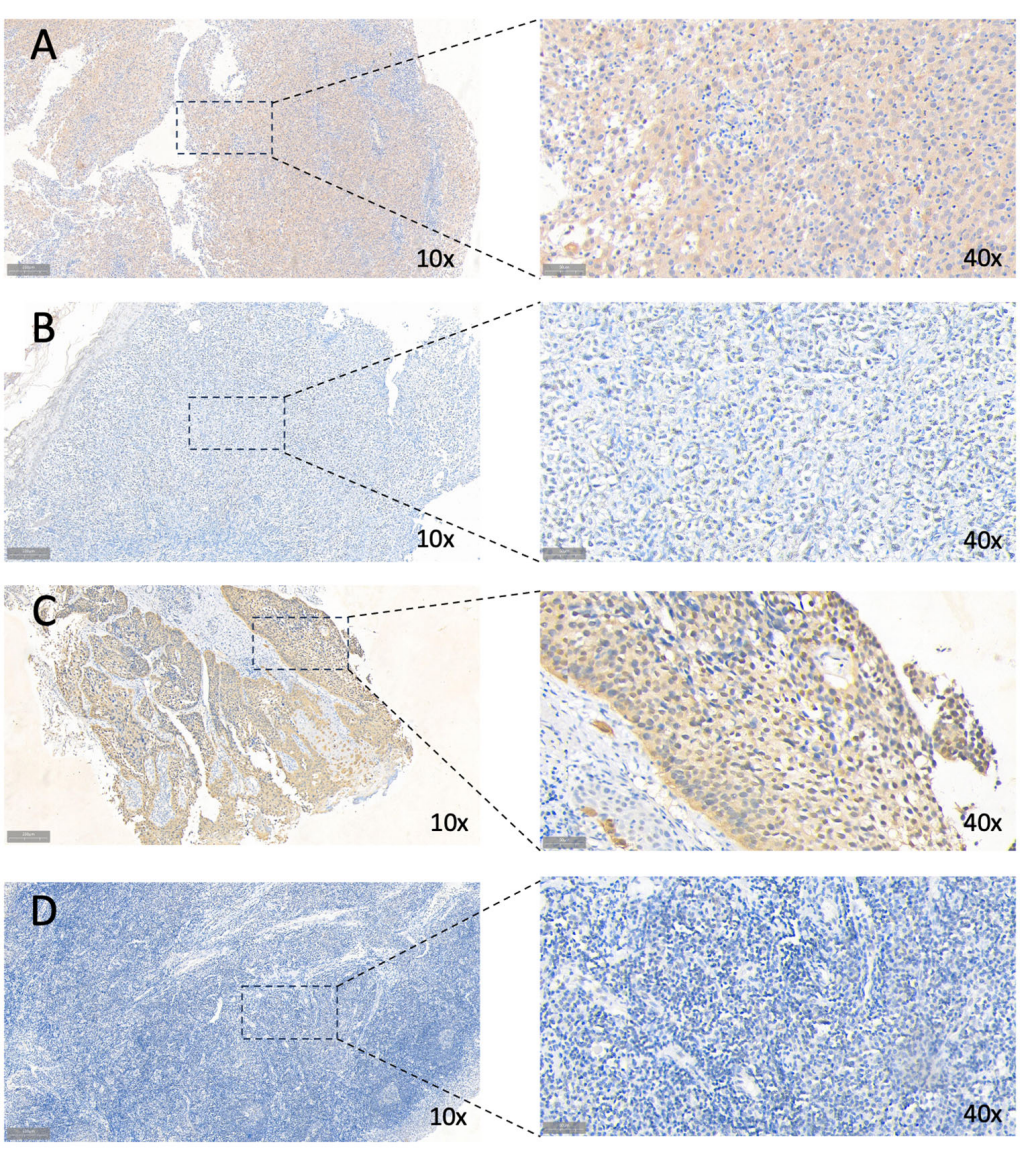
Immunohistochemical staining for TDO2 and P16. **(A)** High TDO2 expression in OPSCC tissues as indicated by IHC. **(B)** Low TDO2 expression in OPSCC tissues as indicated by IHC. **(C)** High P16 expression in OPSCC tissues as indicated by IHC. **(D)** Low TDO2 expression in OPSCC tissues as indicated by IHC. 10× scale at 200μm; 40× scale at 50μm.

Survival analysis was executed on seven hub genes using the Kaplan-Meier method applied to the GSE65858 dataset (**Supplementary Figures 3A–G**). According to the median gene expression, patients were divided into two groups: high expression and low expression level. Notably, the expression levels of BASP1, TDO2, and ZDHHC9 demonstrated a close association with overall survival, indicating an unfavorable prognosis. However the expression of the 7 genes had no effect on disease-free survival (**Supplementary Figures 4A–G**). Subsequent exploration of expression variations in these genes across diverse HPV statuses uncovered a significant divergence in the expression of TDO2 and ZDHHC9 between patients with HPV-positive and HPV-negative oropharyngeal squamous cell carcinoma, with lower expression observed in HPV-positive individuals (**Figure 8A**). Validation with the TCGA cohort affirmed the adverse prognosis linked to TDO2 (**Figure 8B**). Furthermore, pathway analysis revealed a substantial enrichment of TDO2 in biological pathways such as African trypanosomiasis, drug metabolism, extracellular matrix (ECM), and glycosaminoglycan biosynthesis (**Figure 8C**), hinting at a potential association with metabolic pathways under hypoxia. Immune infiltration analysis exposed an increased presence of M0 and M2 macrophages, along with neutrophils, in samples exhibiting high TDO2 expression. In contrast, memory B cells and monocytes were more prevalent in samples with low TDO2 expression (**Figure 8D**). We utilized 46 specimens of oropharyngeal squamous cell carcinoma collected from The First Hospital of China Medical University. Immunohistochemical staining, combined with semi-quantitative scoring, identified 22 cases (47.82%) with high P16 expression as HPV-positive, while 22 cases (47.83%) exhibited high TDO2 expression (**Figures 9A–D**). Statistical analysis revealed a tendency towards lower TDO2 expression in HPV-positive patients (**Supplementary Table 1**). Subsequent analysis of TDO2 expression and clinical data, including gender, age, smoking, alcohol consumption, clinical stage, T stage, and M stage, indicated an association between TDO2 expression and HPV status (P < 0.001) and T stage (P = 0.02).

### 3.5 Analysis of cellular heterogeneity of OPSCC cells

**Figure 10:**
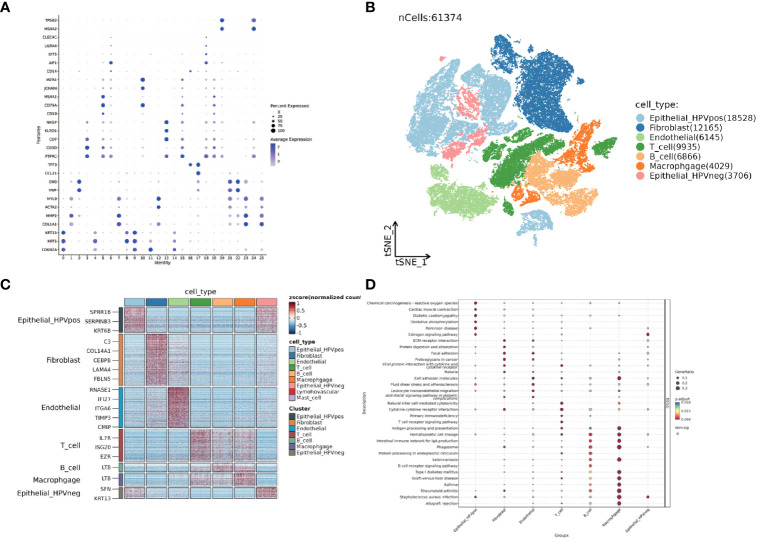
Single-cell dataset GSE182227 TSNE clustering and subgroup functional analysis. **(A)** Dotplot displaying the expression of selected marker genes in 25 subclusters. **(B)** TSNE visualization of seven different major clusters. **(C)** Heatmap of top different expression genes in seven clusters. **(D)** Dotplot shows KEGG analysis of seven clusters. The size of the point represents the GeneRatio and the color represents the adjusted p-value.

The TSNE clustering was performed on 61,347 cells by Seurat package. The results showed that these cells could be clustered into 25 subgroups. The cell markers for annotation are shown in **Figure 10A**. Based on the existing literature reports, combined with the identification and annotation of specific marker genes, they were further classified into seven clusters (**Figures 10B, C**). To deeply understand the biological properties of these subpopulations, we employed the 'SCP' package for KEGG pathway analysis(**Figure 10D**). The results of the analysis revealed the specific biological pathways of different subpopulations. HPV-positive epithelial cells were mainly enriched in pathways such as chemical carcinogens and cardiac contraction; HPV-negative epithelial cells were enriched in pathways related to estrogen receptor; fibroblasts were mainly enriched in extracellular matrix receptor and focal adhesion; endothelial cells showed enrichment in pathways such as cell adhesion molecules and cancer proteoglycans; T cells were enriched in viral protein-cytokine interactions, primary immunodeficiency, and other pathways; B cells showed specific enrichment in hematopoietic cell lines, IgA-producing intestinal immune network, and other pathways; and macrophages showed significant enrichment in cell adhesion molecules, B cell receptors, and other pathways. 7 genes from the hypoxia model were identified using the AUCell algorithm for activity in single-cell sequencing data. The method constructs a gene expression ranking for each cell based on an area under the curve (AUC) value. A value of 0.046 was identified as a suitable threshold. Subsequently, we clustered and colored the t-SNE embedded cells according to the AUC score of each cell (**Supplementary Figure 5**).

### 3.6 Scissor algorithm to map epithelial cells and fibroblasts

**Figure 11:**
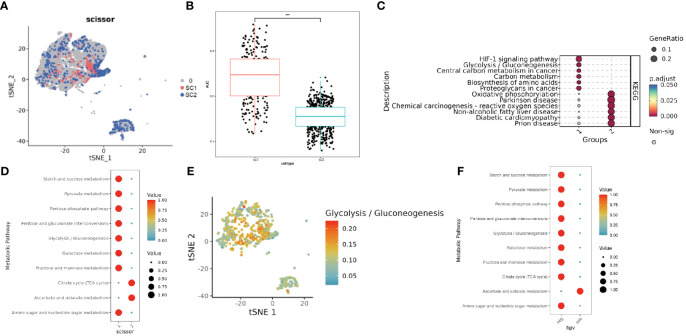
Characterization of two groups of Scissor epithelial cells. **(A)** The Scissor algorithm identifies the two epithelial cells most associated with the two HG groups, SC1 and SC2 cells. **(B)** Hypoxia-related AUC calculated by the AUCell function. **(C)** KEGG analysis in SC1 and SC2 clusters.(1-SC1, 2-SC2). **(D)** Metabolically active pathways analysis in SC1 and SC2 clusters by scMetabolism method (1-SC1, 2-SC2). **(E)** TSNE maps visualize active pathways of Glycolysis. **(F)** Metabolically active pathways analysis in HPV-positive and HPV-negative clusters by scMetabolism method (neg- HPV-negative, pos-HPV-positive). *** means *p*<0.001.

**Figure 12:**
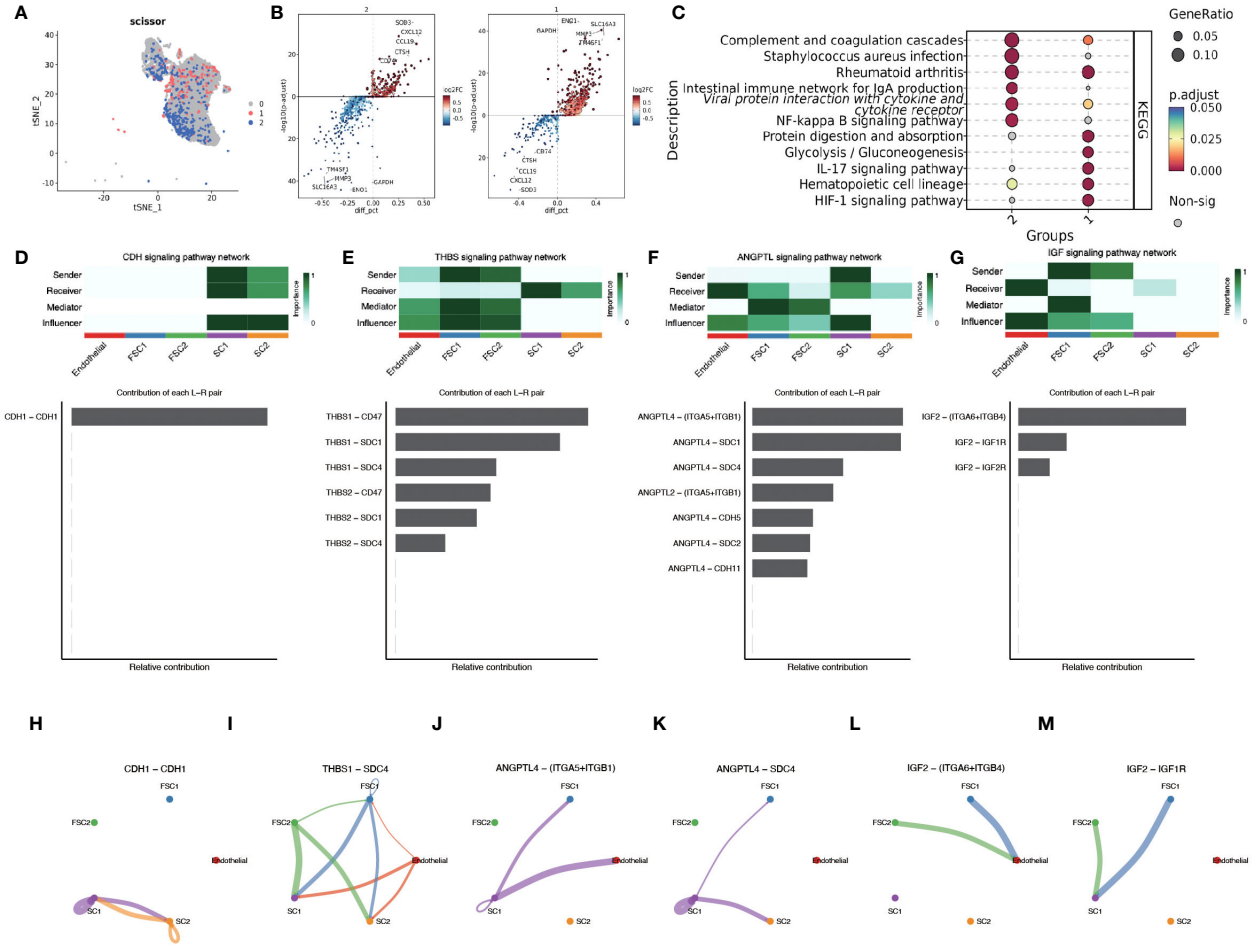
Scissor algorithm for fibroblast clustering and revealing of intercellular crosstalk pathways. **(A)** The Scissor algorithm identifies the two fibroblast cells most associated with the two HG groups defined in GSE171898, FSC1 and FSC2 cells. **(B)** Volcano map showing differential genes between FC1 and FC2. **(C)** KEGG analysis in FSC1 and FSC2 clusters.(1-FSC1, 2-FSC2) **(D, H)** CDH signal network pattern in SC1 and SC2. **(E, I)** THBS signal network pattern in SC1,SC2, FSC1 and FSC2. **(F, J, K)** ANGPTL signal network pattern in SC1,SC2, FSC1 and FSC2. **(G, L, M)** IGF signal network pattern in SC1,SC2, FSC1 and FSC2.

Epithelial cell subpopulations were extracted using the “subset” function, and the scissor algorithm was applied to combine the different HG phenotypes obtained from bulk sequencing of GSE71898 with the single-cell sequencing data to identify two major epithelial cell subpopulations, SC1 and SC2 (**Figure 11A**). Performing AUCell hypoxia score analysis on these two scissor subpopulations, we found that the SC1 cell subpopulation displayed a higher degree of hypoxia (**Figure 11B**). Differential gene analysis (**Supplementary Figure 6**) revealed that the Scissor1 mainly highly expressed genes related to hypoxic stress and metabolism, such as N-MYC downstream regulated genes NDRG1 and lactate dehydrogenase A (LDHA); whereas the SC2 highly expressed intermediate germline kinin (MDK) genes related to epithelial mesenchymal transition (EMT) and genes of nucleosome binding protein HMG family. KEGG analysis revealed that the SC1 was mainly enriched in metabolic pathways such as the HIF-1 pathway and glycolysis, whereas the SC2 subpopulation was mainly enriched in pathways such as oxidative phosphorylation, Parkinson’s disease, and chemical carcinogens (**Figure 11C**). scMetabolism analysis showed that SC1 had higher metabolic activity (**Figures 11D, E**). The level of metabolism also varies by HPV status, with energy metabolic pathways such as glycolysis being stronger in HPV-negative and amino acid metabolism being stronger in HPV-positive (**Figure 11F**). These findings suggest a corroborative relationship between the results of large-scale sequencing analysis and single-cell sequencing analysis. Cell communication analysis using CellChat revealed a greater number and intensity of effects between SC1 and SC2 subpopulations and fibroblasts (**Supplementary Figure 7A, B**). Heatmaps showed that the CDH pathway exhibited strong output signals in the SC1 and SC2 subpopulations, interacting with endothelial cells (**Supplementary Figures 7C, D**). Then we identified two major clusters by scissor, FSC1 and FSC2 (**Figures 12A, B**), of which the FSC1 subpopulation is involved in pathways such as ribosomes and systemic lupus erythematosus. Considering that the main role of fibroblasts in connective tissues is the synthesis of collagen and other extracellular matrix proteins, the activity of ribosomes in such cells may be closely related to the synthesis of these proteins. In contrast, the FSC2 subpopulation is involved in pathways such as transcriptional dysregulation and inflammatory factors in cancer (**Figure 12C**).

### 3.7 Unraveling cellular interaction networks in OPSCC: implications for metastasis and tumor progression

To deeply investigate the cellular interaction network in the immune environment of OPSCC, we used CellChat to reveal the changes in the communication between endothelial, FC1,FC2,SC1 and SC2. CDH signaling showed the highest activity in SC1 and SC2 (**Figures 12D, H**). The autocrine mechanism of CDH mediates direct cell-to-cell interactions through homology-selective adhesion, which influences cell behavior. Thrombospondins (THBS) as a group of multifunctional extracellular matrix proteins, which are potent anti-angiogenic factors. We observed that the interaction between THSB1 secreted by FSC1, FSC2 cell clusters and SCD4, a surface protein of SC1,SC2 cell clusters, promoted intercellular adhesion (**Figures 12E, I**).

We also found that angiopoietin-like family of proteins (ANGPTL) signaling plays a significant role in intercellular crosstalk (**Figures 12F, J, K**). The hypoxic microenvironment promotes the secretion of ANGPTL4. Binding of ANGPTL4 to endothelial cells disrupts their connectivity, increases pulmonary capillary permeability, and promotes vascular endothelial migration of tumor cells. Members of the ANGPTL exhibit autocrine and paracrine activities at different stages of angiogenesis, inflammation, and regulation of cancer progression and metastasis, and in particular the pro-oncogenic role of the C-terminal structural domain of ANGPTL4 has been demonstrated in patients with esophageal squamous cell carcinoma (ESCC) and oral squamous cell carcinoma (OSCC). SDC4, an acetyl asparagine sulfate proteoglycan that controls a variety of cellular processes such as endocytosis, proliferation, and adhesion, and influences signaling pathways such as FGF and VEGF by acting as a co-receptor that binds to a variety of growth factors and extracellular matrix components. There is a paucity of experimental studies on the ANGPTL4-SDC4 ligand receptor, but previous studies have demonstrated that binding of nANGPTL4 to SDC4 can inhibit WNT signaling by decreasing the lysosomal degradation of lipoprotein receptor-associated protein 6. In a study of sclerosing gastric carcinoma, Koichi et al. found that HIF-1α-induced ANGPTL4 could inhibit WNT signaling by up-regulating c -Myc and down-regulation of p27 to promote tumor growth and development of peritoneal metastasis. Therefore, in our study, HPV infection leading to infiltration of the immune environment with E7 promoting HIF-1α expression caused SC1 cell clusters to exhibit activation of ANGPTL4-SDC4 signalling which may be involved in oropharyngeal carcinogenesis and development.

IGF signaling was detected in FC cells clustered with endothelial and SC cells (**Figures 12G, L, M**). Insulin-like growth factor 2 (IGF2) is not only a major factor in the development of primary tumors, but also plays a crucial role in cancer spread, immune evasion, and treatment resistance (20). Activation of the IGF2-Id1-IGF2 loop stimulates chronic aberrant IFN signaling in cancer cells, leading to a stem cell-like phenotype and chemotherapy resistance (21). In breast cancer studies, bidirectional regulation between autocrine IGF2 and inhibitor of DNA-binding 1 (Id1) promotes tumor stemness (22). In contrast, paracrine IGF2 is associated with EMT. Cancer-associated fibroblasts (CAF) from invasive tumors secrete IGF2, which is due to the release of β fibroblast growth factor (βFGF) and transforming growth factor β (TGFβ) from epithelial tumor cells resulting in fibroblasts activation caused by the release of βFGF and TGFβ (23). Activation of AhR by KYN, a product of IDO1 and TDO2, prompts the generation of immune-tolerant dendritic cells (DCs) and regulatory T cells, which together shape a tumor immune microenvironment incapable of recognizing and destroying cancer cells (24). Interestingly, this was also correlated in the TDO2 screened in the previous part of our study. The above findings suggest that SC and FSC cell clusters have metastatic invasive properties and display a more intensely malignant phenotype.

### 3.8 SCENIC to analyze transcription factor activity in SC1 and SC2 cells

SCENIC is an advanced computational method for simultaneously reconstructing gene regulatory networks and identifying cellular states based on single-cell RNA sequencing data. The top five differentially expressed key transcription factors between the SC1 and SC2 cell subpopulations were identified (**Supplementary Figure 8**). Among the SC1 subpopulations, the transcriptional activities of JUNB, ELF1, FOSB, and JUN were particularly prominent. JUNB is an important component of the AP-1 complex, which is commonly activated during stress responses, immune responses, and cell differentiation. ELF1, on the other hand, can regulate a wide range of immune-related genes, including those involved in T cell activation, cytokine production, and B cell function (25). In addition, JUN plays a role in regulating cellular stress responses and inflammatory responses, for example, THBS1 expression is mediated by JUNB on the MAPK pathway. Li et al. (26) showed that tumor glycolytic metabolism may promote immune escape by coordinating the molecular networks of autophagy and CEBPB. CEBPB can bind to the promoter region of TDO2, and the two are highly enriched in mesenchymal subtypes of malignant gliomas, which are associated with a poor prognosis.

## 4 Discussion

**Figure 13:**
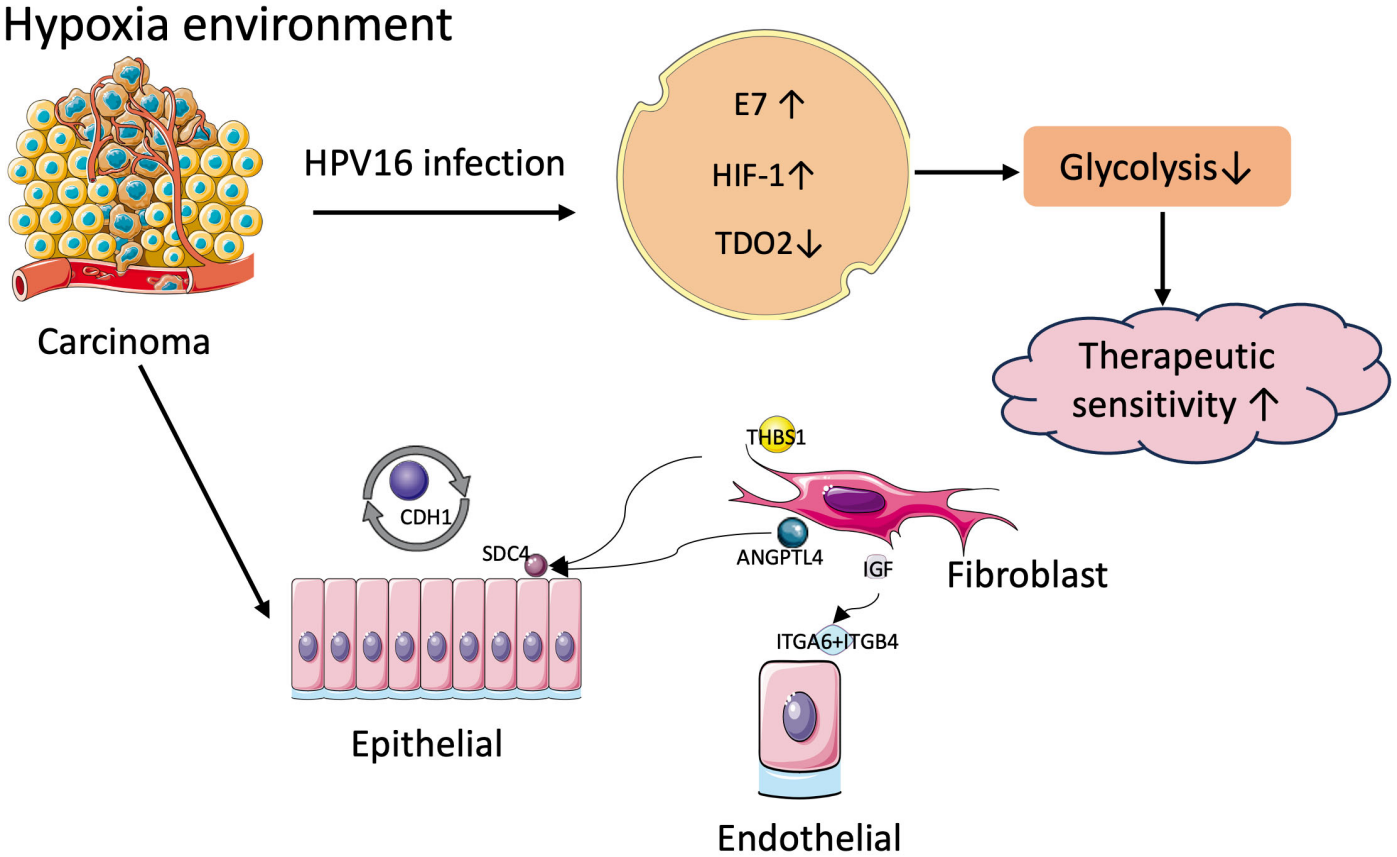
Mechanisms of tumor malignancy in hypoxic environments.

Hypoxia is highly common in most solid tumors, driving malignant progression as well as resistance to radiotherapy and chemotherapy (27). However, the relationship between hypoxia and another important oncogenic factor in HNSCC, HPV infection, has not been clearly elucidated. In this study, we chose the subtype of HNSCC most affected by HPV infection, oropharyngeal cancer, as the study population, and categorized OPSCC patients into two hypoxia-associated subtypes based on the expression of hypoxia-associated genes using concordant clustering. To explore the mechanism of differences in different subgroups, key genes were identified using WGCNA, and further genes related to prognosis were extracted using Lasso-Cox’s method to obtain 7 genes to compose the model. Correspondence between different HPV statuses and hypoxia groups and risk scores were examined by Sankey plots. HPV positive OPSCC patients were found to have lower hypoxia and metabolic degree and better prognosis. Survival analysis and differential expression of each of the seven genes within the model revealed that TDO2 was significantly associated with prognosis, was a risk factor and was lowly expressed in HPV-positive patients. We collected additional clinical samples to validate the predictions using immunohistochemical verification and TDO2 expression was found to be low in HPV-positive oropharyngeal cancer patients. TDO2, a dioxygenase enzyme containing heme, catalyzes the initial step of the kynurenine pathway (KP), specifically the transformation of tryptophan into formyl-kynurenine (28). HIF serves as a significant controller of oxygen homeostasis, managing the equilibrium between oxidative and glycolytic metabolism. HIF triggers the transcription of numerous genes, orchestrating malignant biological processes such as angiogenesis, epithelial-mesenchymal transition, extracellular matrix remodeling, glucose and lipid metabolism, immune evasion, invasion, and metastasis (29). Previous studies have shown that HPV16 E7 protein enhances the transcriptional activity of HIF-1α (13) and HIF-1α negatively regulates TDO2, so we hypothesized that the interaction between these molecules may have a role in prognosis (**Figure 13**) (30).

TDO2 has been identified in colorectal cancer to activate the Kyn-AhR pathway, increase glycolysis to drive metabolic cancer cell growth and CXCL5 secretion to recruit macrophages into the tumor microenvironment (31).We hypothesize that in HPV-positive OPSCC, E7 transcriptional regulation of HIF-1α inhibits TDO2 expression, thereby inhibiting the glycolytic pathway, resulting in a better prognosis and better treatment responsiveness in HPV-positive OPSCC. This needs to be verified by further cellular experiments. IDO1 and TDO2 are the initial Trp metabolizing enzymes of KP and are highly expressed in a variety of cancers (32). The results of the Phase I/II ECHO-204 trial demonstrated that epacadostat, in combination with navulizumab, controlled progression in the HNSCC cohort (33). Dual inhibitors of IDO1 and TDO are also in developmental studies. Wu et al. propose dual IDO1/TDO2 inhibition to enhance anti-tumor immunity in platinum-resistant non-small cell lung cancer (34). In esophageal cancer, TDO2 can upregulate IL-8 through the AKT/GSK3β pathway, thereby inducing polarization of M2 macrophages in ESCC (35). Inhibition of TDO2 may also currently being explored strenuously for their effects in cancer treatment but have not yet reached the clinical trial stage. Both hypoxia and immune infiltration are key features of the tumor microenvironment. By CIBERSORT analysis we found that memory B cells and CD8+ T cells, which are immune-promoting antitumor cells, infiltrated lower in the high-risk group, whereas immunosuppressive tumor-promoting M0 and M2 macrophages were higher. The TIDE score, T-cell dysfunction score and exclusion score of the high-risk score group were significantly increased compared with the low risk. Previous studies have also shown that immunotherapy is less effective in patients with higher levels of hypoxia. HIF signaling also upregulates PD-L1 expression (36), so targeting key downstream effectors of hypoxia or HIF-1α itself has therapeutic advantages in preclinical tumor models. In HNSCC TME, hypoxia suppresses the immune response by inhibiting immune cells, regulating CAF, promoting tumor cell growth, and mediating immune escape (37). HIF proteins and TGF-β promote each other in a positive feedback loop. TGF-β activates and recruits myofibroblasts and fibroblasts in primary tumors and transforms them into CAF (38). The mRNA expression of other CAF-related immunosuppressive regulators (e.g., VEGF, IL6, IL10, and PD-L1) is also markedly enhanced under hypoxic conditions (39).

Subsequently, we mapped the hypoxia phenotype to the single-cell level using a scissors algorithm, identifying SC1 and SC2 subpopulations of epithelial, and FCS1 and FSC2 subpopulations of fibroblasts. AUCell and scMetabolism analyses revealed a higher degree of hypoxia and metabolism in SC1, and HPV-negative epithelial cells were characterized more in favor of the SC1 subtype. CelllChat identified several important cellular pathways. CDH acts predominantly between epithelial cells and mediates tumor progression through adhesion. the THBS pathway is predominantly related to JUNB transcription. We also identified JUNB activity in SC1 subpopulations using the SCENIC algorithm. Several studies support the role of THBS in carcinogenesis, possibly promoting laryngeal cancer cell proliferation and invasion through fatty acid metabolic pathways (40). HIF-1α has been shown to directly upregulate ANGPTL4 expression (41), promoting transendothelial migration and increasing angiogenesis. ANGPTL4 also activates the glycolytic pathway to energize the cell and stimulate EMT, thereby promoting metastasis and chemoresistance (13, 42). IGF2 is predominantly secreted by CAFs, whereas IGF1R is predominantly expressed by cancer cells. IGF1R expression is significantly increased in colorectal cancer tissues and acts as a potent receptor in response to IGF2 stimulation leading to adverse outcomes (43). Similar studies have been performed in breast cancer, where the IGFs/IGF-1R axis causes activation of breast epithelial malignant cells and conversion of stromal fibroblasts to CAFs, and it promotes TME remodeling for tumor invasion (44).

Our study sheds light on the potential clinical implications of TDO2 expression in HPV-positive head and neck cancer patients, including its role in patient stratification, biomarker development, treatment personalization, and mechanistic understanding. Further validation and clinical translation of our findings are necessary to realize the full potential of TDO2 as a prognostic biomarker and therapeutic target in HPV-positive head and neck cancer. Due to the small sample size included in the study, extrapolation to the full population is not yet possible, and the emergence of sequencing studies with larger samples is expected in the future.

## 5 Conclusion

The molecular characteristics of HPV-positive oropharyngeal cancers allow for better prognosis and treatment responsiveness, and quality of life remains high after treatment. Current ongoing clinical trials are also focusing more on life-enhancing treatments while maintaining a robust prognosis. In recent times, the Food and Drug Administration (FDA) has granted approval for two immune checkpoint inhibitors, namely pembrolizumab and nivolumab, in the treatment of recurrent or metastatic head and neck squamous cell carcinoma. In summary, through bioinformatics analysis and immunohistochemistry, we hypothesize the involvement of HIF-1a in regulating TDO2 gene expression and downstream glycolytic pathways in HPV-positive oropharyngeal squamous cell carcinoma. Hypoxia and metabolism are hallmark pathways influencing treatment responsiveness in head and neck cancer, closely linked to HPV status and treatment sensitivity. Our study sheds light on elucidating the mechanisms underlying these treatment response disparities, offering insights into future therapeutic prospects for head and neck cancer. In an increasingly patient-centered clinical environment, personalized treatment and stratified therapy hold promising avenues for further research.

## Data availability statement

The original contributions presented in the study are included in the article/**Supplementary Material**. Further inquiries can be directed to the corresponding author.

## Ethics statement

The studies involving humans were approved by the Ethics Committee of the First Hospital of China Medical University [No (2022).199]. The studies were conducted in accordance with the local legislation and institutional requirements. The human samples used in this study were acquired from primarily isolated as part of your previous study for which ethical approval was obtained. Written informed consent for participation was not required from the participants or the participants’ legal guardians/next of kin in accordance with the national legislation and institutional requirements.

## Author contributions

YZ: Conceptualization, Data curation, Formal Analysis, Methodology, Visualization, Writing – original draft. JY: Investigation, Resources, Validation, Writing – review & editing. CZ: Project administration, Supervision, Writing – review & editing. BZ: Resources, Supervision, Writing – review & editing.
