# Supplementary material for: Establishment of a prognostic model for hypoxia-associated genes in OPSCC and revelation of intercellular crosstalk
Source: Front Immunol. 2024 Jun 3;15:1371365. doi: 10.3389/fimmu.2024.1371365 (PMC11181350; doi:10.3389/fimmu.2024.1371365)
Supplement: Supplementary file 1 [file DataSheet_1.docx]

Supplementary Material

# Supplementary Figures and Tables

## Supplementary Tables

| **Variable** | **Total** | **TDO2 Expression N (%)** | | **P-value** |
| --- | --- | --- | --- | --- |
|  |  | **TDO2-up** | **TDO2-down** |  |
| **No. patients** | 46 | 22 (47.83) | 24 (52.13) |  |
| **Gender** |  |  |  | 0.486 |
| Female | 7 (15.21) | 2 (28.57) | 5 (71.43) |  |
| Male | 39 (84.79) | 20 (51.28) | 19 (48.72) |  |
| **Age** |  |  |  | 0.331 |
| ≥60 | 20 (43.48) | 9 (45.00) | 11 (55.00) |  |
| <60 | 26 (56.52) | 13 (50.00) | 13 (50.00) |  |
| **Smoking** |  |  |  | 0.840 |
| No | 10 (21.74) | 4 (40.00) | 6 (60.00) |  |
| Yes | 36 (78.26) | 18 (50.00) | 18 (50.00) |  |
| **Alcohol usage** |  |  |  | 0.857 |
| No | 11 (23.91) | 5 (45.45) | 6 (54.54) |  |
| Yes | 35 (76.09) | 17 (48.57) | 18 (51.43) |  |
| **P16 expression***** |  |  |  | 0.003 |
| Up | 22 (47.83) | 4 (18.18) | 18 (81.82) |  |
| Down | 24 (52.17) | 18 (75.00) | 6 (25.00) |  |
| **Stage** |  |  |  | 0.425 |
| I-II | 13 (28.26) | 5 (38.46) | 8 (61.54) |  |
| III-IV | 33 (71.74) | 17 (51.52) | 16 (48.48) |  |
| **T stage*** |  |  |  | 0.018 |
| T1-T2 | 17 (36.96) | 12 (70.59) | 5 (29.41) |  |
| T3-T4 | 29 (63.04) | 10 (34.48) | 19 (65.52) |  |
| **N stage** |  |  |  | 0.887 |
| N0-N1 | 33 (71.74) | 16 (48.48) | 17 (51.52) |  |
| N2-N3 | 13 (28.26) | 6 (46.15) | 7 (53.84) |  |

**Supplementary Table 1. Summary of clinical data for all patients in the present cohort (n = 46). The identification of HPV status was based on the immunohistochemical results of P16, where high expression of P16 was defined as HPV-positive. Significance between clinical variables and TDO2 expression was analyzed using the chi-square test .(* means *P* < 0.05, *** means *P*<0.001.)**

| **Variable** | **Number(%)** |
| --- | --- |
| **Age** |  |
| >60 | 36(40.45) |
| ≤60 | 53(59.55) |
| **Gender** |  |
| Male | 74(83.15) |
| Female | 15(16.85) |
| **HPV status** |  |
| Negative | 46(51.69) |
| Positive | 43(48.31) |
| **Stage** |  |
| I-II | 12(13.48) |
| III-IV | 77(86.52) |

**Supplementary Table 2.** Clinical characteristic of GSE65858.

| **Variable** | **Number(%)** |
| --- | --- |
| **Age** |  |
| >60 | 18(25.71) |
| ≤60 | 52(74.29) |
| **Gender** |  |
| Male | 61(87.14) |
| Female | 9(12.86) |
| **HPV status** |  |
| Negative | 26(37.14) |
| Positive | 44(62.86) |
| **Stage** |  |
| I-II | 16(22.86) |
| III-IV | 54(77.14) |

**Supplementary Table 3.** Clinical characteristic of TCGA-OPSCC.


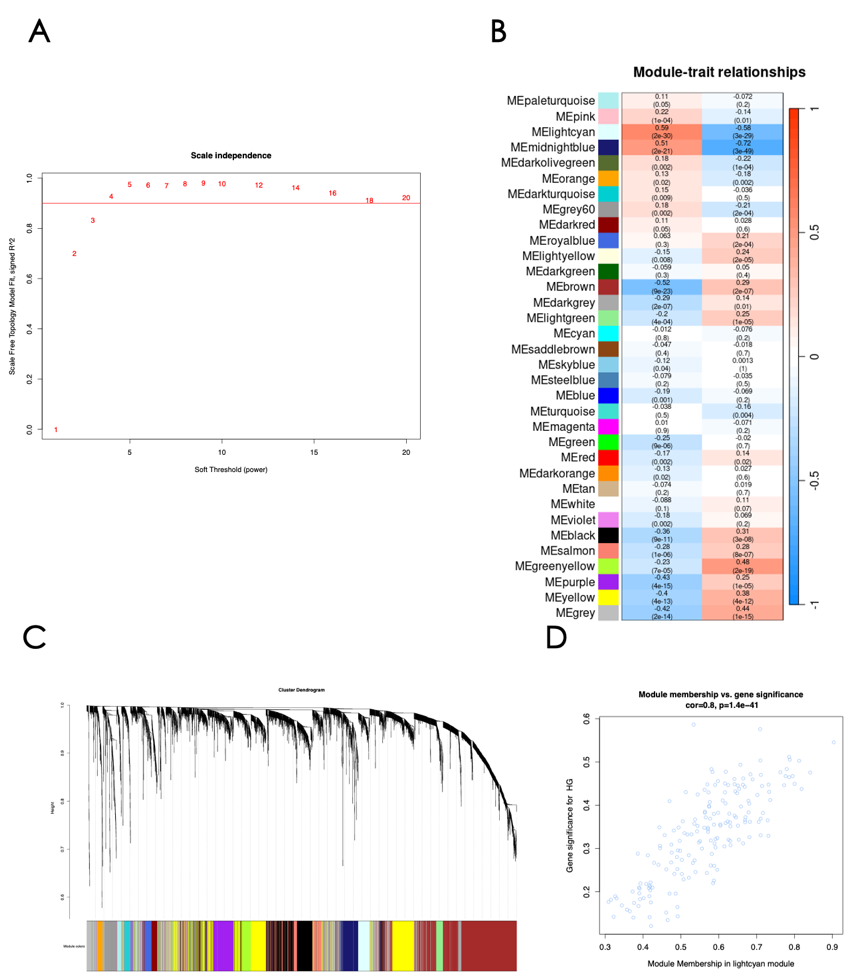


**Supplementary Figure 1.**

**(A)** WGCNA Optimal Threshold Selection.

**(B)** Analysis of correlations between the modules and hypoxia group, p.values are shown.

**(C)** Cluster dendrogram of the co-expression network modules (1-TOM)

**(D)** Scatterplot of the most correlated lightcyan modules (*cor=0.8, p<0.05*).

**
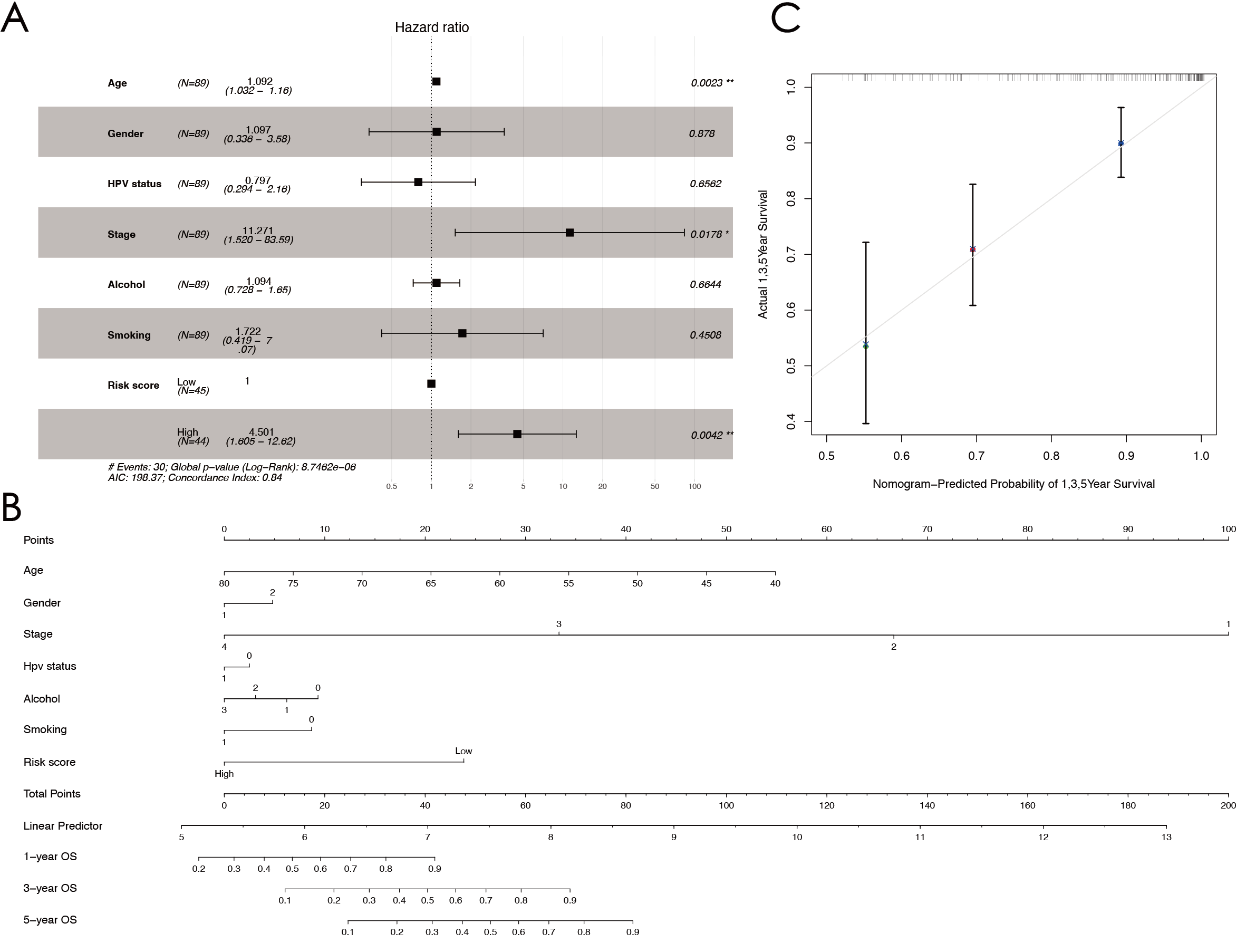
**

**Supplementary Figure 2.**

**(A)** Multivariate Cox regression analysis of significant clinical characteristics and risk scores.

**(B)** The nomogram constructed with clinical information and risk model to predict the 1-, 3-, and 5-year OS in the GSE65858 cohort.

**(C)** Fitting curves for evaluating the predictive efficacy of nomogram.


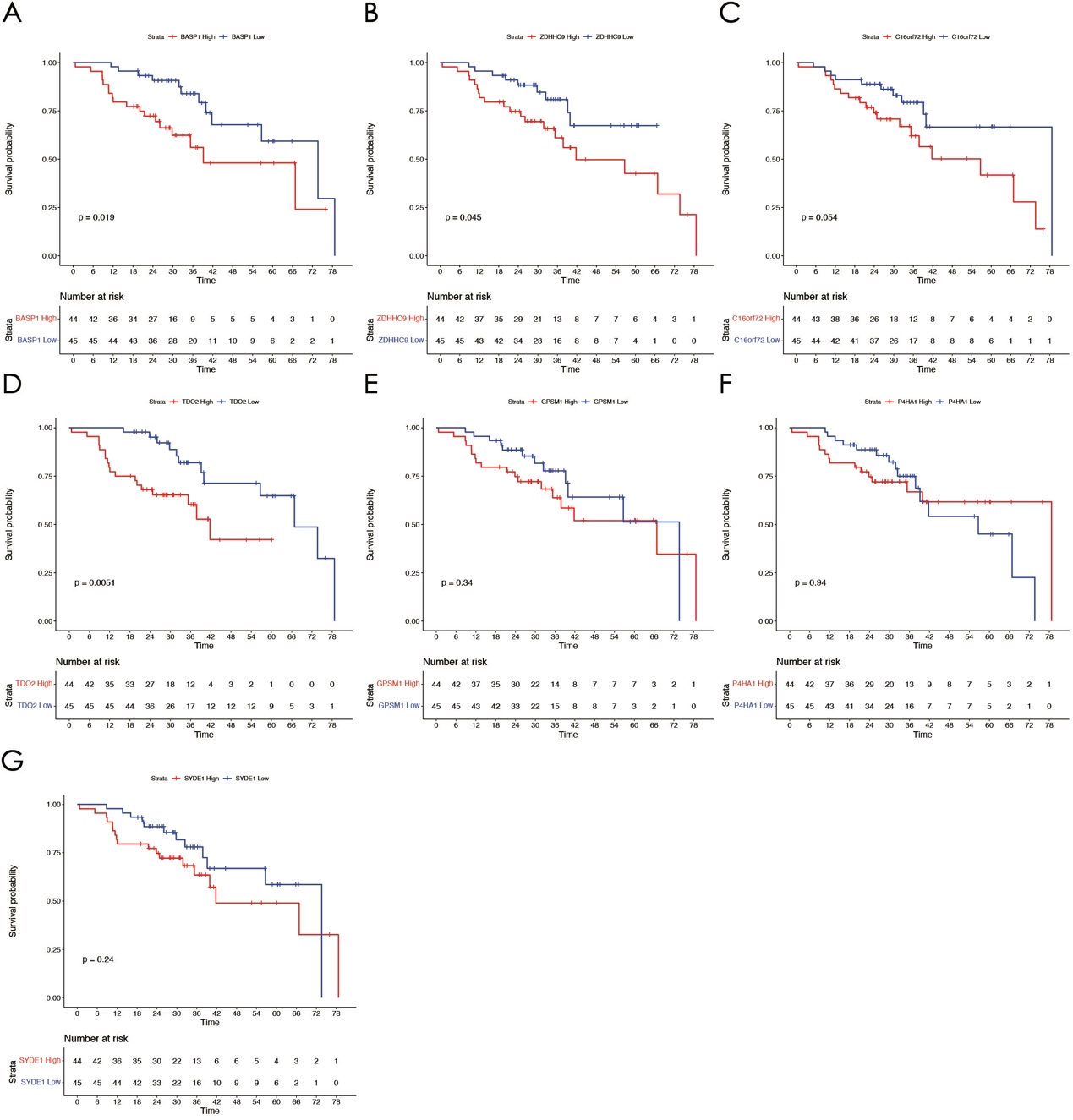


**Supplementary Figure 3.**

**(A)-(G)** Survival curves for OS(Overall Survival) for 7 key genes in the GSE65858 cohort. (BASP1, ZDHHC9, C16orf72, TDO2, GPSM1, P4HA1, SYDE1)

**
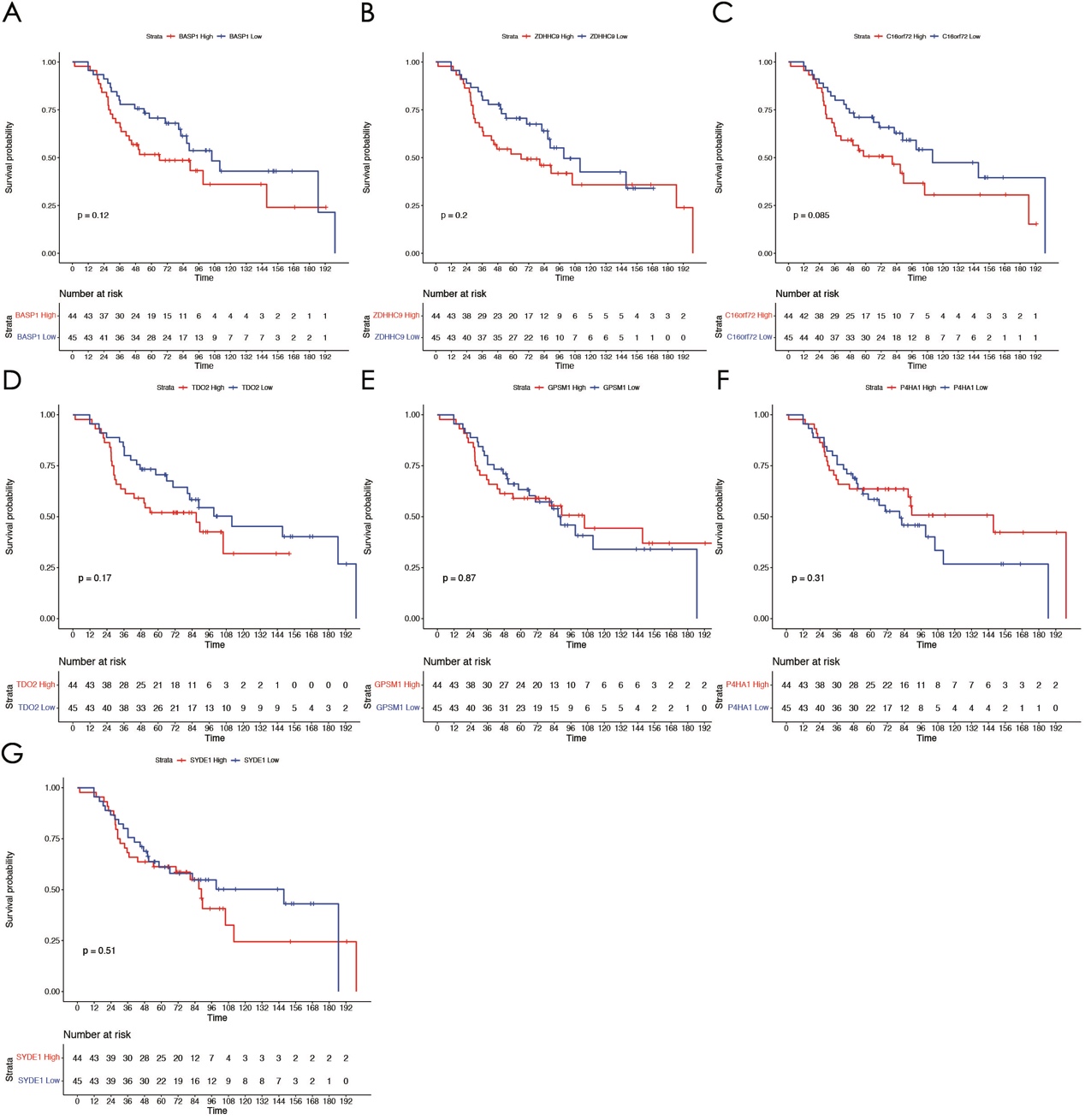
**

**Supplementary Figure 4.**

**(A)-(G)** Survival curves for DFS (disease-free survival) for 7 key genes in the GSE65858 cohort. (BASP1, ZDHHC9, C16orf72, TDO2, GPSM1, P4HA1, SYDE1)

**Supplementary Figure 5.** AUCell identifies hypoxia-associated genes.


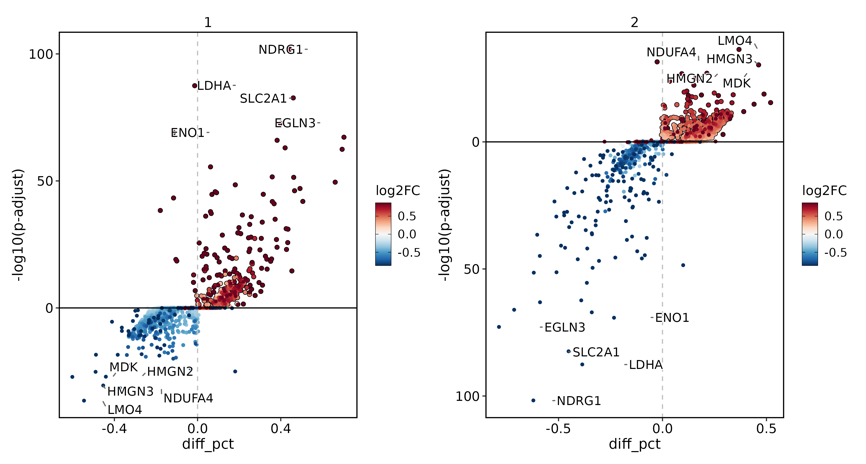


**Supplementary Figure 6.** Volcano map of SC1 and SC2 differential genes.

**
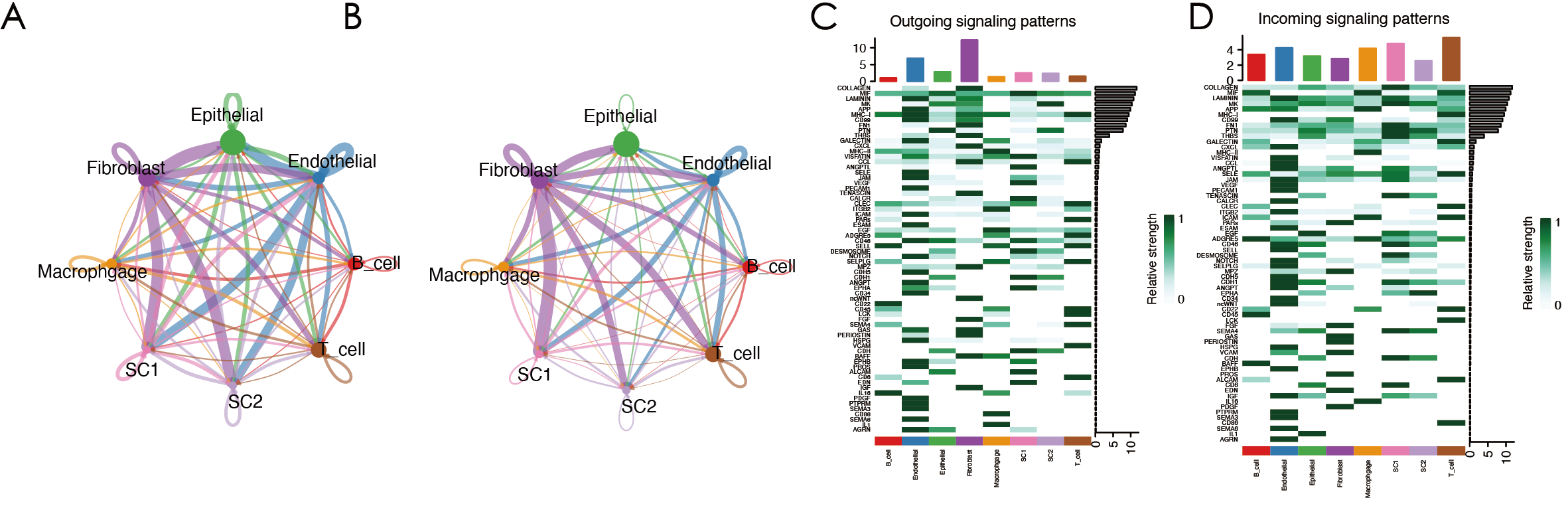
**

**Supplementary Figure 7**

**(A)** Diagrams displaying the interaction number in cell clusters.

**(B)** Diagrams displaying the interaction strength in cell clusters.

**(C),(D)** Heatmap visualizing the overall signaling pathway contribution strength between different cell subpopulations.


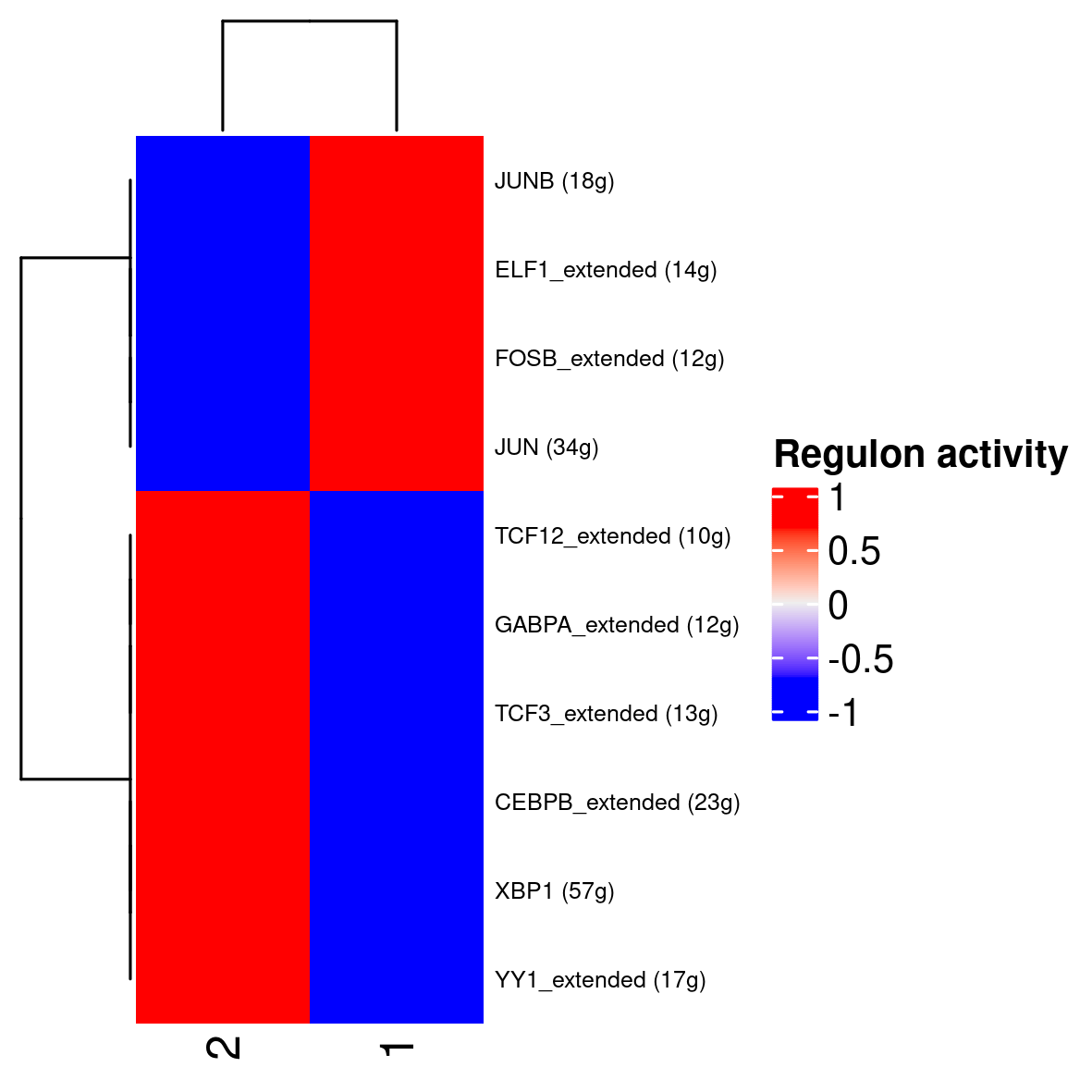


**Supplementary Figure 8.** Analysis of SC1 and SC2 transcription factor activity by SCENIC.
